# Augmented Reality and Artificial Intelligence for the Assessment and Rehabilitation of Spatial Neglect: A Systematic Review

**DOI:** 10.1177/15459683261445440

**Published:** 2026-05-04

**Authors:** Shaojun Li, Benjamin Chong, Ghazal Mehri-Kakavand, Catherine Shi, Denise Taylor, Allan Fowler, Mark Billinghurst, Monika Harvey, Alan Wang

**Affiliations:** 1 Auckland Bioengineering Institute, University of Auckland, New Zealand; 2 Faculty of Medical and Health Sciences, University of Auckland, New Zealand; 3 Department of Data Science & AI, School of Engineering, Computer & Mathematical Sciences, Auckland University of Technology, New Zealand; 4 School of Clinical Sciences, Auckland University of Technology, New Zealand; 5 Faculty of Engineering and Design, University of Auckland, New Zealand; 6 School of Psychology and Neuroscience, University of Glasgow, UK; 7 Medical Imaging Research Center, Faculty of Medical and Health Sciences, The University of Auckland, New Zealand; 8 Centre for Co-Created Ageing Research, The University of Auckland, New Zealand; 9 Centre for Brain Research, The University of Auckland, New Zealand

**Keywords:** spatial neglect, augmented reality, artificial intelligence, stroke rehabilitation, ecological validity, personalized neurorehabilitation

## Abstract

**Background and Purpose:**

Augmented reality (AR) and artificial intelligence (AI) have been applied to the assessment and rehabilitation of post-stroke spatial neglect (SN). This study aims to evaluate the feasibility, effectiveness, degree of personalization, and ecological validity of AR, AI, and hybrid methods for SN assessment and rehabilitation.

**Methods:**

PubMed, Scopus, Web of Science, Embase, CINAHL, IEEE Xplore, and the ACM Digital Library were searched up to August 2025. Two reviewers independently screened articles, extracted data, and assessed risk of bias and outcome-level certainty.

**Results:**

Of 268 screened studies published between 2000 and 2025, 15 met the inclusion criteria, including 11 assessment studies (8 AI, 1 AR, and 2 hybrid) and 4 AR rehabilitation studies, involving 567 participants. AI assessment methods demonstrated high diagnostic accuracy (area under the curve (AUC) up to 0.95), and 1 AR assessment showed strong diagnostic accuracy (AUC = 0.89). Four AR rehabilitation studies reported acceptable feasibility, with 1 randomized controlled trial (RCT) showing improvements in several neglect outcomes. Ecological validity and personalization were generally very low, and the overall certainty of evidence ranged from low to very low.

**Conclusion:**

Current evidence for AR and AI SN assessment and rehabilitation methods remains insufficient to determine their feasibility, effectiveness, ecological validity, and degree of personalization, largely due to small sample sizes, methodological heterogeneity, and the limited number of RCTs. Future research should focus on developing standardized, scalable frameworks that integrate AR with adaptive AI models, and multicenter RCTs are required to confirm clinical efficacy and long-term functional outcomes.

## Introduction

Following a stroke, spatial neglect (SN) typically presents as a severe neurocognitive impairment characterized by an inability to attend to or interact with stimuli in the space contralateral to the cerebral injury.^1^ It affects approximately 30% to 38% of people with acute stroke, while its chronic phase prevalence remains uncertain due to inconsistent evidence.^2,3^ SN is a clinically heterogeneous condition encompassing multiple overlapping behavioral subtypes, including peripersonal, extrapersonal, allocentric, motor, representational, and personal neglect. These may occur individually or in combination within the same individual.^4^ Nevertheless, neuropsychological and neuroimaging evidence support the presence of a homogeneous core deficit underlying these manifestations. This is characterized by a systematic bias of attention toward the ipsilesional side and reduced processing of contralesional stimuli.^5,6^ The functional expression of this core deficit varies substantially across individuals, time course, and arousal levels,^7^ resulting in diverse and often persistent impairments that significantly impede functional recovery, prolong hospitalization, and reduce the likelihood of achieving independent living after discharge.^8^

Accurate identification of SN through behavioral signs is essential for clinical decision making and rehabilitation planning. However, no gold standard assessment method exists in clinical practice.^9-11^ Current validated approaches include paper-and-pencil, behavioral, and comprehensive batteries.^9^ Paper-and-pencil tests (eg, line bisection, cancellation, and figure copying) are widely used for their simplicity and low cost.^12^ Nevertheless, they show poor sensitivity to mild or subtype-specific neglect and are affected by ceiling effects.^12^ Behavioral tests, such as the Kessler Foundation Neglect Assessment Process, involve structured observation of daily activities (eg, dressing, eating, and navigation) to improve diagnostic accuracy.^12,13^ However, the time-consuming nature and the requirement for professional training limit their clinical feasibility.^13^ The Behavioral Inattention Test (BIT),^14^ recommended by international guidelines,^15^ integrates paper-and-pencil subtests with simulated daily tasks (eg, menu reading and telephone dialing) to balance diagnostic accuracy and clinical feasibility. Despite this integrative design, it remains insensitive to specific neglect subtypes, and its simulated tasks may not adequately reflect real-world performance.

Compared with assessment methods, SN rehabilitation methods remain less standardized.^10^ Methods show considerable variability in intervention types, treatment frequency, follow-up duration, and outcome measures.^16,17^ Non-invasive rehabilitation approaches typically include visual scanning training,^18^ prism adaptation,^19^ visuomotor feedback training,^20^ and mirror therapy.^21^ Although these interventions often yield short-term improvements, Longley et al^22^ reported that the certainty of evidence remains low, and translation to real-world functional recovery is limited. Moreover, these approaches require therapist supervision, and their repetitive and monotonous nature may reduce engagement and adherence in people with SN.^23,24^

With advances in technology, augmented reality (AR) and artificial intelligence (AI) offer potential opportunities for developing more automated, ecologically valid, and engaging SN assessment and rehabilitation methods. AR overlays virtual objects onto the real world in real time, enabling safe, controlled, and repeatable rehabilitation within real physical environments.^25^ Mixed reality (MR) extends this concept by spatially anchoring virtual content and managing depth and occlusion through scene-aware behaviors, providing a more immersive and interactive experience.^26^ As AR and MR are often used interchangeably in practice, this review collectively refers to them as AR. Existing reviews have reported positive effects of AR interventions on upper and lower limb recovery after stroke, suggesting that AR interventions may facilitate better transfer of training to daily activities.^27,28^

AI, particularly machine learning (ML) and deep learning (DL), can analyze complex behavioral and neurophysiological data to support more accurate assessment and personalized rehabilitation,^29^ thereby supporting clinicians in decision-making and potentially reducing manual workload. Recent reviews have emphasized its potential in acute and post-stroke rehabilitation.^30,31^ Moreover, a review has shown that integrating AI with virtual reality (VR)/AR technologies can further improve the adaptability and efficacy of remote rehabilitation.^32^

To our knowledge, no systematic review has evaluated AR, AI, or their integration in the assessment or rehabilitation of SN. Given the persistent challenges in SN assessment or rehabilitation, synthesizing existing AR and AI studies can help clarify their applicability and provide clinicians with more personalized, automated, and ecologically valid alternatives or complementary tools. Therefore, this review aims to synthesize current evidence on how AR and AI technologies have been applied to SN, evaluating their feasibility, effectiveness, degree of personalization, and ecological validity. In addition, we identify methodological limitations and outline future directions for integrating AR and AI to advance SN assessment and rehabilitation.

## Methods

### Protocol and Registration

The systematic review was performed in accordance with the Preferred Reporting Items for Systematic Reviews and Meta-Analysis (PRISMA) 2020 guidelines.^33^ The study was registered with the International Prospective Registry of Systematic Reviews: CRD420251115777.

### Data Sources and Search Strategy

To identify AR and AI studies in the assessment and rehabilitation of SN, we searched the following scientific databases: PubMed, Scopus, CINAHL, Web of Science, Embase, IEEE Xplore, and the ACM Digital Library. The search was on 12 August 2025. We used both controlled vocabulary (eg, Medical Subject Headings (MeSH) and terms) and free-text keywords, structured around 3 core concepts: (1) SN, (2) technology-based interventions, and (3) clinical relevance. Boolean operators (AND/OR) and truncation symbols were used to enhance search comprehensiveness. The full search strings used for each database are provided in the Supplemental Appendix 1.

Titles, abstracts, and full texts were independently screened by 2 reviewers (SL) and (GMK) to identify AR and/or AI studies in SN assessment or rehabilitation. Any discrepancies were resolved through discussion or, when necessary, by consultation with a third reviewer (AW). We also tracked citations and searched relevant conference proceedings to ensure comprehensive coverage.

### Eligibility Criteria

We applied the Participants, Interventions, Comparisons, Outcomes, and Study designs framework to formulate the inclusion and exclusion criteria.

#### Participants

We included studies with adults clinically diagnosed with SN after stroke, regardless of chronicity or lesion severity. Studies comparing adults with SN with healthy controls were also eligible, while those involving only healthy participants or people with stroke without SN were excluded.

#### Interventions

We included studies that employed AR technologies, comprising AR-capable hardware (eg, head-mounted displays or handheld devices) and associated software applications, as part of the intervention or assessment design. Studies that employed AI technologies (eg, ML, DL, large language models, or neural networks) in the assessment or rehabilitation process design were also included, regardless of the hardware platform used. We excluded studies that did not incorporate either AR or AI technologies in the SN assessment or rehabilitation process, such as those only involving VR, robotic devices, or other unrelated digital interventions.

#### Comparisons

We included studies comparing AR and/or AI assessments with conventional paper-and-pencil and/or behavioral tests, and those evaluating pre–post outcomes or comparing AR and/or AI rehabilitation with standard therapist-led interventions (eg, visual scanning training, limb activation, prism adaptation, and task-specific exercises).

#### Outcomes

For assessment studies, outcomes included diagnostic performance (sensitivity, specificity, and AUC) compared with conventional methods. For rehabilitation studies, the primary outcome was the change in SN severity from baseline to post-intervention (and follow-up, if available), measured by clinically validated scales, neurophysiological outcomes, or standard paper-and-pencil tests. Additional outcomes included changes in functional performance (eg, Barthel Index and Functional Independence Measure) and usability or acceptability measures (eg, System Usability Scale, Simulator Sickness Questionnaire, task completion rate, and adverse events).

We also evaluated the degree of personalization and ecological validity in AR and/or AI assessment and rehabilitation methods. As no standardized metric exists for personalization, we applied the taxonomy of Figueiredo et al^34^ to classify studies across 5 dimensions: rule-based systems, biofeedback, behavioral tracking, context-awareness, and intelligent monitoring. Based on this framework, personalization was categorized as low (behavioral monitoring or rule-based adjustments), moderate (multiple adaptive components such as behavioral tracking with biofeedback or context-awareness), or high (intelligent monitoring, eg, individualized modeling or adaptive decision-making). Ecological validity, defined as the extent to which experimental paradigms reflect real-world performance contexts, was assessed using the Ecological Validity Assessment Tool.^35^ This tool evaluates how closely assessment and rehabilitation approaches align with real-world conditions across the environment, stimulus, response, body, and mind dimensions.^35^

#### Study Designs

We included both randomized controlled trials (RCTs) and non-RCT (non-RCTs) published on or after 1 January 2000 to capture studies conducted during the development of modern AR and AI systems.

### Data Extraction

Two reviewers (SL and GMK) performed data extraction independently. Any disagreements were settled by discussion or, where required, decided by a third reviewer (AW). Extracted items included: bibliographic and study design information (title, authors, year, country, study design, and objectives); sample and stroke characteristics (sample size, controls if applicable, age, sex, recruitment/representativeness, time since stroke/stroke phase, lesion laterality, and anatomical site, baseline severity such as National Institutes of Health Stroke Scale [NIHSS]/modified Rankin Scale [mRS]); the tests and reference standards used to classify SN and their thresholds (and how diagnostic accuracy was defined, eg, sensitivity/specificity/AUC); intervention details (design, dose, and comparator content), outcomes and assessment timepoints (baseline, immediate post-intervention, and follow-up); AR/AI technical details and validation strategies (device/platform, data modalities, model type, and cross-validation/external validation); usability/implementation metrics (setup/training time, assessment duration, System Usability Scale [SUS], Simulator Sickness Questionnaire [SSQ], or other user experience [UX] measures, adverse events); personalization-related features (eg, rule-based systems, biofeedback, behavioral tracking, context-awareness, and intelligent monitoring) and ecological validity dimensions (environment, stimulus, response, body, and mind); and the main findings and conclusions.

### Risk of Bias (RoB) Assessment and Certainty of Evidence

The RoB of the included studies was assessed using design-specific tools appropriate to each study type. RCTs were evaluated using the Cochrane RoB-2) tool,^36^ while non-randomized intervention studies were assessed using the RoB in Non-randomized Studies of Interventions tool.^7^ Single-case experimental designs were appraised using the Single Case Design RoB tool.^37^ Diagnostic studies were evaluated using the Quality Assessment of Diagnostic Accuracy Studies-2 (QUADAS-2) framework.^38^ RoB judgments were assigned according to the guidance of each respective tool.

The certainty of evidence across key outcome domains was evaluated using a narrative adaptation of the Grading of Recommendations Assessment, Development and Evaluation (GRADE) framework.^39^ Certainty ratings started as high for RCTs and low for non-RCTs, with upgrading permitted only in exceptional cases. Certainty was rated down across the 5 GRADE domains (RoB, inconsistency, indirectness, imprecision, and publication bias). Because of heterogeneity and limited quantitative data, a narrative synthesis was conducted, providing a single outcome-level certainty rating per outcome (high, moderate, low, or very low) with explicit reasons for each downgrade or upgrade. Two reviewers (SL and GMK) independently assessed study quality and RoB, resolving disagreements through consensus or, when necessary, adjudication by a third reviewer (AW).

### Data Synthesis

Due to the substantial heterogeneity in the study designs, AR devices, AI algorithms, outcome measures, and reporting formats of the included studies, a quantitative meta-analysis was not feasible. Instead, we conducted a structured narrative synthesis following the PRISMA 2020 guidelines.^33^

## Results

### Search Process

**Figure 1.**
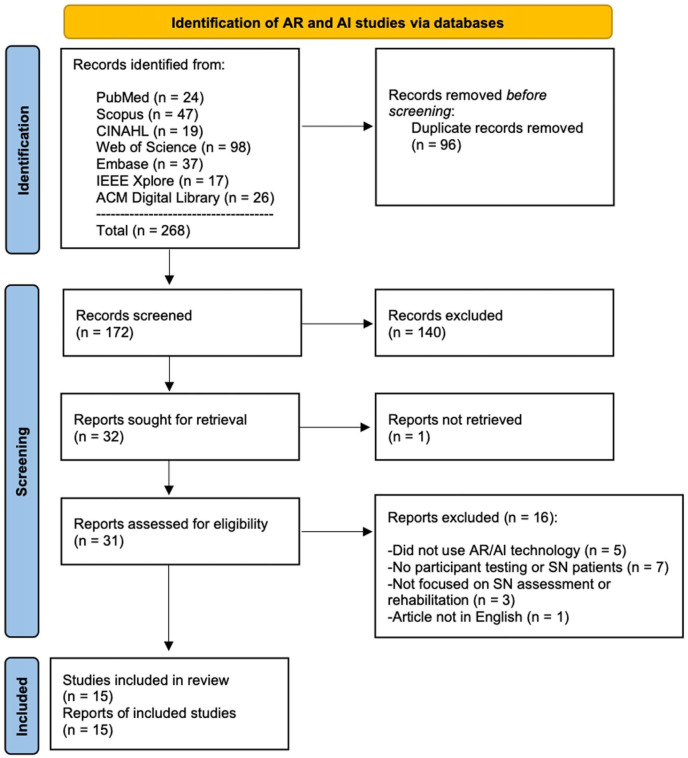
Preferred Reporting Items for Systematic Reviews and Meta-Analyses 2020 flow diagram.

The initial search identified 268 articles. After removing 96 duplicates and excluding 140 records based on title and abstract screening, 32 articles were retrieved for full-text review. In total, 15 studies met the inclusion criteria.^40-54^ Among the included studies, 11 studies focused on SN assessment, including 8 AI studies,^47-54^ 1 AR study,^46^ and 2 studies integrating both AR and AI technologies.^40,42^ The remaining 4 studies are all AR SN rehabilitation studies.^41,43-45^ The PRISMA 2020 flow diagram is presented in Figure 1.

### Characteristics of Included Studies and Participants

Between 2020 and 2024, 15 studies were conducted in Germany (n = 5), the United States (n = 3), 1 each from the United Kingdom, Belgium, Italy, Switzerland/France (collaboration), the Republic of Korea, Japan, and the Netherlands. Eight studies adopted cross-sectional diagnostic or case–control designs,^47-54^ while 5 were feasibility or pilot investigations, including a single-case design and a design-oriented pilot study.^40-43,45^ The remaining 2 studies comprised 1 RCT^44^ and 1 cross-sectional diagnostic accuracy study.^46^

As summarized in Table 1, the included AR studies involved 67 participants with SN, 6 stroke participants without SN, and 45 healthy controls, and the AI studies included 149 participants with SN, 234 stroke participants without SN, and 66 healthy controls. Across both groups, the mean age of participants with SN typically ranged from the late 50s to early 60s (overall mean ≈ 61 years), with a male predominance (approximately 65%-70%). Most people with SN were in the subacute phase (7 days-3 months after stroke), whereas non-neglect stroke controls were predominantly chronic (>6 months). Right-hemisphere lesions were most frequent among participants with SN, and both ischemic and hemorrhagic strokes were represented, although etiology and lesion lateralization were inconsistently reported. Detailed lesion mapping was rare, except in the studies by Stammler et al.^43,44,46^

**Table 1.** Characteristics of the Included Rehabilitation and Assessment Studies.

Study (country)	Design	Technology and purpose	Sample (n, age, sex)	Stroke stage/lesion	SN diagnosis/control	ECOVAL score
Mak et al^40^ (US)	Pilot	AR + AI; Assessment	SN 5 (2 M/3 F), WSN 5 (4 M/1 F); SN 61.4 ± 18.6 y, WSN 54.4 ± 23.1 y	Early subacute–chronic; R/L mixed	BIT-C < 129; groups by BIT-C	2
Takazawa et al^41^ (JP)	Feasibility (single case)	AR; Rehabilitation	n = 1 (L USN, M 52 y)	Subacute (124 d post-onset)	CBS as outcome; no control	8
Kocanaogullari et al^42^ (US)	Feasibility	AR + AI; Assessment	SN 5 (2 M/3 F), WSN 5 (3 M/2 F); SN 67.4 ± 11.5 y	Acute–chronic; R/L mixed	BIT-C; groups by neglect severity	2
Stammler et al^43^ (DE)	Feasibility	AR; Rehabilitation	10 patients (8 M/2 F), 10 healthy; mean 61 ± 15 y	Subacute–chronic; R lesions	≥ 2/4 tablet tests positive	7
Stammler et al^44^ (DE)	RCT	AR; Rehabilitation	Negami 10 (8 M/2 F), Standard 10 (6 M/4 F); mean 61 y	Subacute–chronic; R lesions	Same cut-offs; groups matched	7
Bakker et al^45^ (NL)	Design-based	AR; Rehabilitation	VSN 7 (patients); OTs 8; other HCPs 7; sex/age NR	Geriatric rehab facilities	CBS or hospital neuropsych tests	8
Stammler et al^46^ (DE)	Cross-sectional	AR; Assessment	Stroke: n = 20 (15 male, 5 female), age 59.6 (14.6) years; Healthy controls: n = 20 (10 male, 10 female), age 60.1 (13.4) years.	Post-stroke 145 d; R lesions	≥ 2/4 tablet tests positive	8
Liang et al^47^ (UK)	Case–control	AI + Tablet; Assessment	Training: SN 33 + Ctr 110; Testing: SN 19 + Ctr 27; Age >60; Sex NR	Mixed post-stroke stage	BIT < 130 = SN (4-level scale)	2
De Boi et al^48^ (BE)	Case–control	AI + VR; Assessment	HC 15 (10 M/5 F), N– 13 (7 M/6 F), N+ 10 (4 M/6 F); mean ≈ 63 y	Mixed stroke types; R lesions	≥ 2/3 positive (BHT, LBT, VSTT)	5
Donisi et al^49^ (IT)	Case–control	AI; Assessment	Stroke 35 (USN 11, non-USN 24); 20 M/15 F; mean 59.9 ± 14.5 y	NR; R 19/L 16	Clinical labels (USN vs non-USN)	2
Kocanaogullari et al^50^ (US)	Case–control	AI; Assessment	SN 5 (3 M/2 F), WSN 6 (3 M/3 F); SN 64.6 ± 10.4 y	Acute–subacute; R/L mixed	BIT < 129 or subtest cut-offs	2
Kim et al^51^ (KR)	Diagnostic	AI + VR; Assessment	SN 19 (14 M/5 F), non-SN 22 (17 M/5 F), HC 22 (10 M/12 F); mean ≈ 50 y	Chronic ≥ 3 mo; R lesions	LBT < 7; SCT < 51; CBS >1	6
Franceschiello et al^52^ (CH/FR)	Diagnostic study	AI + Eye-tracking; Assessment	SN 15 (9 M/6 F), HC 9; mean ≈ 58 y (45-69)	Chronic (≈306 d); R lesions	Bells, Letter, Line Bisection, Drawing, Reading	5
Rosenzopf et al^53^ (DE)	Case–control	AI; Assessment	R stroke 20 (N 12: 8 M/4 F; non-N 8: 7 M/1 F); mean ≈ 64 y	Acute–chronic; R lesions	Letters & Bells + Copying (≥ 2 abnormal)	2
Belger et al^54^ (DE)	Case–control	AI + VR; Assessment	SN 20 (15 M/5 F), non-SN 19 (10 M/9 F), HC 20 (13 M/7 F); mean 59 ± 9 y	Chronic; R lesions	CBS + NET; age comparable	6

Abbreviations: SN: spatial neglect; WSN: without spatial neglect; CBS: Catherine Bergego Scale; NIHSS: National Institutes of Health Stroke Scale; mRS: modified Rankin Scale; NR: not reported; R/L: right/left hemisphere; Bilat: bilateral; VSN: visuospatial neglect; OT: occupational therapist; HCP: healthcare professional; MoCA: Montreal Cognitive Assessment; FIM: Functional Independence Measure; BI: Barthel Index; CoC: center-of-cancellation; EWB: end-weighted bisection; NET: neglect evaluation battery; SCT: star cancellation test; LBT: line bisection test; BHT: baking tray task; VSTT: visual search training task; ECOVAL: Ecological Validity Assessment Tool; BIT-C: Behavioral Inattention Test–Conventional. Stroke stages: hyperacute (0-24 hours), acute (1-7 days), early subacute (7 days-3 months), late subacute (3-6 months), and chronic (>6 months).

Sampling was predominantly convenience-based from single-center rehabilitation facilities or institutional databases, with consecutive recruitment reported only in a minority of studies.^44,53^ Diagnostic criteria for SN varied widely across studies, including the Behavioral Inattention Test–Conventional (BIT-C) in AR-based EEG-guided neglect detection system (AREEN) and electroencephalography (EEG) studies,^40,42,50^ tablet-based cutoffs for center-of-cancellation or egocentric weighting bias,^43,44,46^ the Catherine Bergego Scale (CBS) or prior neuropsychological tests,^45,51^ and composite paper-and-pencil batteries such as line bisection, star cancellation, and visual scanning tasks.^48,52-54^ Stroke severity measures (eg, NIHSS and mRS) were reported only in Stammler’s RCT.^44^

### Assessment Applications

#### AR Assessment Applications

As shown in Table 2, the Free Exploration Test (FET) developed by Stammler et al^46^ on a tablet-based AR platform (iPad) was the only AR assessment application identified. Participants freely explored a visual environment without explicit cues in this task, and their exploration trajectories were quantified. With an average testing time of approximately 3 minutes, the mean horizontal exploration-bias angle effectively discriminated participants with SN from controls, demonstrating strong diagnostic validity (AUC = 0.89; sensitivity = 0.85; and specificity = 1.00). The FET also showed moderate correlations with conventional paper-and-pencil tests (Letter Cancellation: *r* = .56; Bells Test: *r* = .49).

**Table 2.** Characteristics and Key Findings of the Included AR Rehabilitation and Assessment Studies.

Study	Device/platform	Task paradigm	Feedback modality	Duration/frequency	Key outcomes
Mak et al^40^	HoloLens 1 + EEG + MATLAB	AR “Starry Night” search with clicker	None (assessment)	Two sessions	AUC ≈ 0.83; *r* = .81 with BIT-C; neglect map visualized.
Takazawa et al^41^	HoloLens 2 + Unity	Object search with walking	Audio chime + visual ball + feedback	20 min daily × 2 weeks	CBS improved (12→6); gaze-left ↑ from 40 to 75%.
Kocanaogullari et al^42^	HoloLens 1 + EEG (16 ch)	AR “Starry Night” visual search with EEG classification	None (assessment)	Single session (~1-2 min fine-tuning)	Accuracy improved from 18.7% to 81.6%; no AEs.
Stammler et al^43^	iPad Pro + ARKit	“Follow bird” and “Find bird” tasks	Visual + auditory cues	20-25 min × 5/wk × 2 wks	High usability (SUS 4.3/5); no AEs.
Stammler et al^44^	iPad Pro + Negami app	AR bird tasks vs. visual scanning training	Adaptive visual + auditory cues	25 min × 5/wk × 2 wks	Negami > standard on 3/5 tests; effects sustained at 1-2 mo.
Bakker et al^45^	HoloLens 1	Virtual museum search task	Audio prompts from neglected side	Single session	Improved scanning and mobility; qualitative feedback.
Stammler et al^46^	iPad Pro + ARKit	Free exploration test	None (assessment)	Single 3 min session	AUC 0.89; sensitivity 0.85; specificity 1.00; no AEs.

Abbreviations: BIT-C: behavioral inattention test–conventional; VST: visual scanning training; AUC: area under the receiver operating characteristic curve; SUS: System Usability Scale; SSQ: Simulator Sickness Questionnaire; AEs: adverse events; NS: not significant.

#### AI Assessment Applications

Tables 2 and 3 show that all AI studies aimed to detect or quantify SN across diverse AI models and hardware platforms. The applied models included linear/logistic regression,^47^ support vector machines (SVMs),^51-54^ tree-based ensembles (eg, Random Forest and Classification and Regression Tree),^49,54^ EEG-specific convolutional neural networks (EEGNet),^50^ and probabilistic Gaussian processes with active learning.^48^ Data sources ranged from digital pen-tablet or touchscreen tasks,^47,53^ EEG,^50^ and immersive VR environments,^48,51,54^ to eye-tracking,^54^ and clinical-record pipelines.^49^ Only Liang et al^47^ reported the assessment duration under 3 minutes, while no studies described the setup time.

**Table 3.** Characteristics and Key Findings of the Included AI Assessment Studies.

Study	Model/technology	Input features	Validation	Hardware/platform	Key outcomes
Mak et al^40^	AR–EEG; Logistic Regression, RDA–KDE	EEG bandpower and CSP features	10-fold CV; 10 pts × 2 sessions	HoloLens + 16-ch EEG	SN detection AUC = 0.83; EEG indices correlated with BIT-C (*r* = .81).
Kocanaogullari et al^41^	EEGNet CNN (fine-tuned per subject)	700 ms EEG epochs (14 ch, 2-60 Hz)	Subject-wise transfer	AREEN AR task + EEG	Accuracy ↑ from 6% to 64%→65% to 99% after fine-tuning (<2 min).
Liang et al^47^	Bayesian linear regression	Pen-tablet kinematics (cancellation and drawing)	Train/test split (33 + 110→19 + 27)	WACOM tablet	Accuracy ≈ 92.5%; assessment < 10 min; strong agreement with BIT.
De Boi et al^48^	Gaussian Process Regression	Eye/head angles, spatial asymmetry metrics	Independent groups; test–retest	Pico Neo 2 Eye VR	AUC > 0.80; reliable retest; VR more sensitive than paper-based tests.
Donisi et al^49^	RF, GB, AdaBoost (clinical data)	MoCA, FIM, BI, lesion site	10-fold CV (N = 35)	KNIME software	RF: Acc = 0.92, Sens = 0.83, Spec = 1.00; lesion site most predictive.
Kocanaogullari et al^50^	EEGNet CNN	700 ms EEG (16 ch, 2-62 Hz)	10-fold CV	AREEN screen task	Acc ≈ 89.7%; Sens ≈ 87%; key sites Cz, P1, F3 informative.
Kim et al^51^	SVM on VR FOPR task	Fixed/free-head RT and accuracy	5-fold CV (subject-wise)	Oculus Rift DK1 VR	Acc ≥ 83%; strong agreement with LBT, SCT, CBS.
Franceschiello et al^52^	1D/2D CNN, SVM, RF (AI gaze models)	Eye-tracking trajectories (300 Hz)	5-fold CV; 10 reps	Tobii TX300 eye-tracker	AUC ≈ 0.85; Acc ≈ 86%-89%; correlated with SLF3 FA and severity.
Rosenzopf et al^53^	SVM (linear/RBF)	Digital cancellation features	Leave-One-Subject-Out CV	27″ touchscreen + stylus	Balanced Acc = 88%-98%; robust across screen sizes.
Belger et al^54^	RF + CART	113 VR behavioral and movement features	10 × 10-fold CV	HTC Vive Pro Eye	Acc = 77% (3-class); head-movement and timing most predictive.

Abbreviations: VSN: visuospatial neglect; USN: unilateral spatial neglect; BHT: Baking Tray Task; BI: Barthel Index; RT: reaction time; Sens: sensitivity; Spec: specificity; AER: average error rate; ROC: receiver operating characteristic; CV: cross-validation; LOSO: leave-one-subject-out; CART: Classification and Regression Tree; RF: random forest; RotF: rotation forest; GB: gradient boosting; DT: decision tree; NB: naïve Bayes; LDA/QDA: linear/quadratic discriminant analysis; RDA: regularized discriminant analysis; KDE: kernel density estimation; MLP: multilayer perceptron; SVM: support vector machine; CNN: convolutional neural network; CSP: common spatial pattern; SMOTE: synthetic minority oversampling technique; SLF3 FA: superior longitudinal fasciculus III fractional anisotropy; EEGNet: EEG-specific convolutional neural networks; FIM: Functional Independence Measure.

Performance across classification tasks was generally strong. EEG-based models achieved high performance in classifying SN from non-SN EEG signals, with an accuracy of around 89.7% and a sensitivity of 87%, indicating informative contributions from parietal and frontal electrodes.^52^ Tablet-based and digital task models demonstrated strong correspondence with standard paper tests, yielding 88% to 98% balanced accuracy within <10-minute assessments.^47,53^ VR and eye-tracking–based systems achieved AUCs exceeding 0.80 and accuracies >83%,^48,51,52,54^ showing good alignment with clinical measures and reliable short-term retest performance. Clinical and multimodal data models reached the highest AUC (0.95) and specificity (1.00) using Random Forests and Gradient Boosting on conventional clinical scores.^49^ Validation across studies relied mainly on internal cross-validation; only 1 study reported a test–retest reliability, and no external or prospective validations were reported.^48^

#### AI and AR Assessment Applications

Mak et al^40^ introduced AREEN, an AR paradigm using a head-mounted display (HoloLens) and the Starry Night visual search task. The system integrated behavioral clicker responses and EEG recordings, which were analyzed using regularized discriminant analysis and kernel density estimation to classify neglect (AUC = 0.83) and predict response speed (AUC = 0.76). Importantly, AREEN generated individualized neglect-field heatmaps that correlated strongly with conventional paper-based measures (BIT-C, *r* = .81). Building on this framework, Kocanaogullari et al^42^ implemented subject-specific fine-tuning of convolutional neural networks (EEGNet), substantially improving per-subject classification accuracy (eg, <20% → >80%) within minutes of calibration, as shown in Table 2 and 3. However, neither study reported the duration of task setup or testing.

### Rehabilitation Applications

Four AR rehabilitation studies were identified, as shown in Table 2. Stammler et al^43^ evaluated the Negami tablet-based AR app. In a feasibility study, participants with SN were trained by holding an iPad in one hand and turning their head and trunk to search for a virtual bird in a physical environment. Training lasted 2 weeks (5 sessions per week, ~25 minutes/session). High usability (SUS >80) with minimal cybersickness was reported. In a subsequent RCT,^44^ 20 participants with SN were randomized to either Negami training or standard visual scanning therapy with comparable dosage (~25 min, 5 times weekly for 2 weeks). Both groups were matched at baseline. Within group analyses showed that Negami training led to significant improvements on 4 of 5 neglect tests (Letter Cancellation, Bells, Copying, Exploration; all *P* < .01), whereas the control group improved only on the Copying Task (*P* < .05). Between group post-intervention comparisons showed significantly greater improvements in the Negami group on 3 measures (Bells, Copying, and Exploration), with benefits maintained at 1- to 2-month follow-up. Takazawa et al^41^ developed a HoloLens 2 system simulating daily object-search tasks with real-time auditory and visual cues for missed targets. A single participant with SN showed clinical improvement (CBS 12 → 6; gaze-left ratio 40.1% → 74.9%) following 20-minute daily sessions over 2 weeks. Bakker et al^45^ created a HoloLens 1 serious game in a virtual museum, combining enhanced visual contrast and same-side auditory prompts to encourage contralesional scanning. Although users and therapists reported high engagement, no standardized outcome measures of neglect severity were provided.

### Ecological Validity and Personalization

As shown in Table 1, 60% of studies fell within the low-to-moderate ecological validity range (≤6), with only a few achieving high validity. Among these, AR rehabilitation methods exhibited higher ecological validity (total score ≥7),^41,43-46^ characterized by a naturalistic training environment, natural body engagement, realistic interaction, multimodal feedback, and low cognitive load. Four AI and 2 hybrid assessment methods demonstrated low ecological validity (total score = 2).^40,42,47,49,50,53^ This was mainly because these AI methods were often digitized versions of traditional paper-based tests, with artificial testing contexts and relatively high cognitive demands.

Overall, the level of personalization across the included studies was low. Of the 15 studies, 11 demonstrated low personalization, relying mainly on rule-based task adjustments or basic behavioral monitoring without adaptive modeling to differentiate individual SN profiles.^40,41,43-47,50,51,53,54^ Three studies showed moderate personalization, primarily among AI-based diagnostic approaches using ML to classify SN based on behavioral, clinical, or physiological features.^42,49,52^ Only 1 study implemented high personalization through an active learning framework based on Gaussian Process Regression to construct individualized spatial attention profiles.^48^

### RoB and Quality Assessment

**Figure 2.**
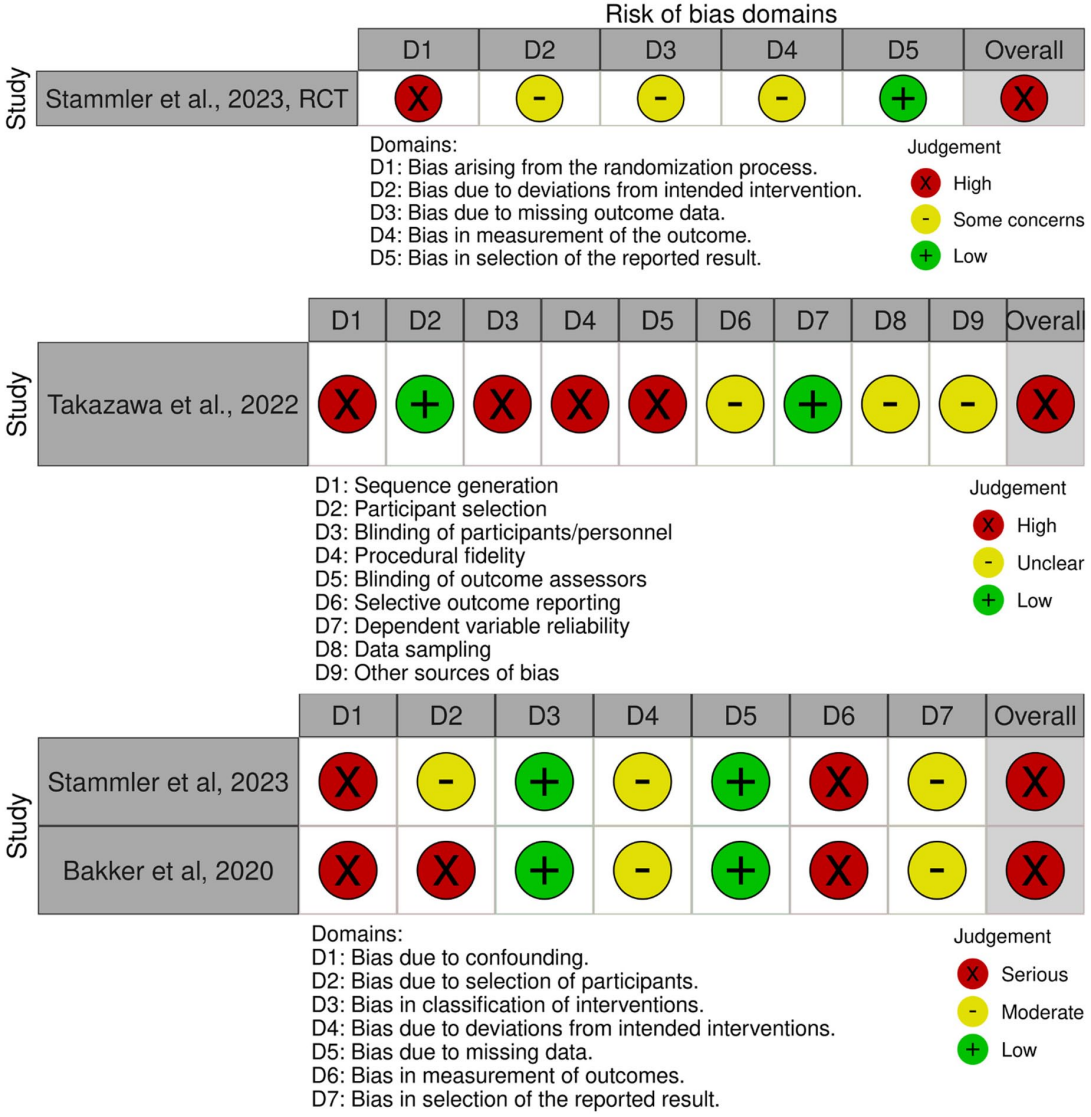
Risk-of-bias assessment of included rehabilitation studies using Risk of Bias 2, Risk of Bias in Non-randomized Studies of Interventions, and the Single Case Design Risk of Bias. Visualization created with robvis.^55^

Figure 2 presents the RoB assessment for the rehabilitation studies. Overall, all rehabilitation studies were judged to have a high or serious RoB. The single-case experimental study was rated as high RoB,^41^ and the 2 non-randomized intervention studies^43,45^ were judged to have serious RoB due to confounding and limitations in outcome measurement. The RCT by Stammler et al^44^ was also rated as high RoB, mainly due to concerns regarding the randomization process and potential selection bias.

**Figure 3.**
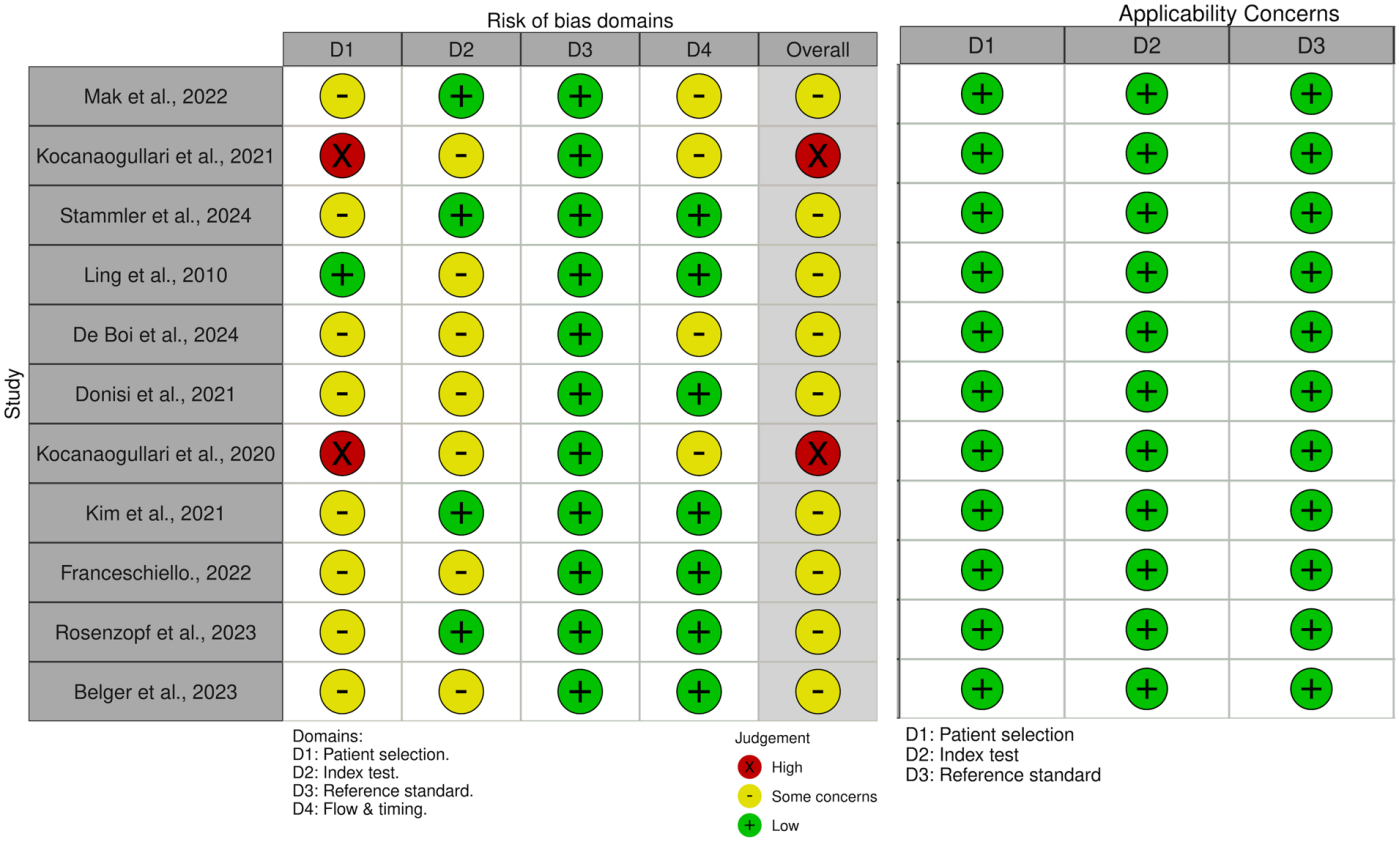
Risk-of-bias and applicability assessment of included assessment studies using Quality Assessment of Diagnostic Accuracy Studies-2. Visualization created with robvis.^55^

As shown in Figure 3, 9 assessment studies were judged to have some concerns regarding RoB,^40,46-49,51-54^ while 2 studies were rated as high risk.^42,50^ The main sources of bias were patient selection and dataset limitations, including small sample sizes, unclear recruitment strategies, and the use of healthy controls without appropriate stroke controls. Additional concerns were related to the index test, as most ML models were evaluated using cross-validation without independent external validation. Despite these limitations, reference standards were generally appropriate across studies, and applicability concerns were judged to be low.

### GRADE Summary of Evidence

Based on the GRADE assessment,^39^ the certainty of evidence across the evaluated outcomes ranged from low to very low. For assessment studies, the certainty of evidence was rated as low. The evidence was limited by indirectness, as most evaluations were conducted in simulated or controlled settings, and by imprecision due to small sample sizes and lack of independent external validation. For rehabilitation studies, the certainty of evidence was rated as very low. The evidence was downgraded due to high RoB across all rehabilitation studies, imprecision arising from very small sample sizes, and indirectness resulting from substantial heterogeneity in intervention protocols and outcome measures. The certainty of evidence for outcomes related to ecological validity and personalization was rated as very low. This downgrade was due to high heterogeneity in study designs, imprecision from small samples and reliance on proxy outcomes.

## Discussion

This review aimed to examine current applications of AR and AI in the assessment and rehabilitation of SN and to evaluate their feasibility, effectiveness, personalization, and ecological validity. Given the substantial heterogeneity across the 15 included studies, a grouped narrative synthesis and GRADE assessment were conducted. Overall, the current evidence remains insufficient to determine their feasibility, effectiveness, ecological validity, and degree of personalization. The narrative synthesis suggests that AI assessment methods demonstrate promising diagnostic accuracy, but evidence regarding their clinical feasibility remains limited, and ecological validity and personalization remain low. AR rehabilitation methods demonstrate acceptable feasibility and relatively high ecological validity, but evidence supporting their clinical effectiveness and personalization remains limited. Small sample sizes, lack of independent external validation, substantial methodological heterogeneity, limited number of RCTs, and the overall high RoB across studies further weaken the current evidence.

### Assessment Methods

Our first narrative synthesis revealed that AR, AI, and hybrid SN assessment methods demonstrated relatively high diagnostic accuracy, but the overall quality of evidence was low, and their real-world clinical feasibility remains uncertain. The tablet-based AR FET represents a rapid and low cognitive load assessment method that can be deployed across various environments, demonstrating high diagnostic accuracy and strong translational potential.^47^ However, its clinical effectiveness still requires validation through RCT. Notably, the method has been used only to distinguish the presence or absence of SN, rather than leveraging individual exploration trajectories to generate personalized neglect profiles. The AREEN system was the only application combining AR and AI for SN Assessment,^40,42^ demonstrating the potential to integrate AR platforms with AI algorithms to enhance assessment performance. However, the study design did not fully exploit the unique advantages of AR in real-world spatial anchoring and natural environmental interaction, instead adapting traditional paper-and-pencil tasks into an AR format. Although including EEG data improved diagnostic accuracy, it substantially increased setup time and limited the system’s scalability in clinical practice.

The included AI applications primarily employed conventional ML models, which consistently achieved high classification accuracy for SN across clinical and behavioral datasets.^49-54^ This trend is consistent with previous reviews on AI-based diagnosis of cognitive impairment.^56^ Compared with DL approaches, these models can be more robust under small-sample conditions and offer greater interpretability. However, most studies were conducted on single-center datasets with limited sample sizes and without external validation, increasing the risk of overfitting and limiting model generalizability. Variations in sample composition and feature extraction strategies may further reduce the comparability of model performance across studies. In addition, most studies did not report setup or testing duration and provided no direct evidence of clinical effectiveness.

Despite promising diagnostic accuracy, new SN assessment methods must maintain high accuracy while ensuring short setup and testing times to minimize clinical workload. In addition, these systems should require minimal professional training and be readily integrated into existing clinical workflows.

### Rehabilitation Methods

Our second narrative synthesis indicated that AR rehabilitation methods generally demonstrated acceptable feasibility, as reflected by high task completion rates, acceptable usability scores, and low reports of adverse effects. However, evidence for therapeutic effectiveness remains insufficient. All methods were grounded in visual scanning training, a well-established neurorehabilitation approach that incorporates visual and auditory cueing and gamification strategies consistent with attention-control models, such as the Corbetta–Shulman framework.^57^ However, despite this solid theoretical basis, only 1 RCT^44^ employed full randomization and assessed both short- and long-term outcomes. Most other studies had methodological limitations, including small sample sizes, non-randomized designs, heterogeneous outcome measures, and limited follow-up, which constrained the interpretability of current findings. Training dosage and quantification were often inconsistent, validation standards were frequently lacking, and spontaneous recovery (except for Stammler et al^44^ where this was explicitly measured and controlled for), could not be fully ruled out in most studies.

At the implementation level, there is no consensus on which AR hardware platform (tablet-based vs. head-mounted) is most suitable for SN rehabilitation. Researchers must balance immersion, usability, cognitive load, and potential adverse effects. No studies have yet implemented AI-driven or combined AR–AI rehabilitation interventions. Evidence for real-world effectiveness in clinical environments is also lacking, and the neurophysiological mechanisms underlying these rehabilitation methods remain unexplored. Future studies should verify the effectiveness of AR rehabilitation through larger RCTs with standardized outcome measures and follow-ups. Comparative evaluations of different AR hardware could identify optimal balances between immersion, usability, and cognitive load. Investigating the neurophysiological mechanisms underlying AR methods will help clarify how behavioral improvements relate to cortical reorganization. Finally, developing standardized frameworks for training dosage and validation metrics will be essential for the clinical scalability and real-world translation of AR rehabilitation methods.

### Personalization and Ecological Validity

Our third narrative synthesis indicated that current AR, AI, and hybrid methods have not yet effectively enhanced the personalization or ecological validity of SN assessment and rehabilitation. Although De Boi et al^48^ and Kim et al^51^ generated neglect heat maps based on individual’s characteristics within VR environments, these outputs were limited to classification purposes and did not provide individualized diagnostic reports or rehabilitation guidance. AI and hybrid methods have incorporated behavioral and physiological data such as eye tracking, EEG, and reaction time, leading to improved diagnostic accuracy.^42,49,52^ However, their potential for distinguishing neglect subtypes and supporting personalized interventions remains largely unexplored. None of the AR rehabilitation methods integrated any form of personalization. Future research should examine how behavioral and physiological signals can be transformed into individualized assessment metrics and integrated into AR rehabilitation methods to deliver adaptive, person-centered training that may enhances rehabilitation outcomes.

Although AR assessment and rehabilitation methods guided people with SN to perform natural visual scanning behaviors in real environments through tablets or head-mounted displays, thereby enhancing realism, stimulus diversity, and embodied interaction,^41,43-46^ these studies were conducted in controlled hospital or rehabilitation settings, which reduced environmental unpredictability and attentional load. Improving ecological validity may facilitate better transfer of training to individual’s daily activities. Therefore, future research should explicitly identify the factors that enhance ecological validity and develop systematic frameworks to evaluate the ecological validity of both assessment and rehabilitation methods.

### Limitations

This review has several limitations that should be acknowledged. First, although a systematic search strategy was employed, the small number of eligible studies limited the strength and breadth of the evidence base. Second, despite careful construction of the search terms, discrepancies in terminology, particularly the inconsistent classification of SN assessment and rehabilitation, may have resulted in the omission of relevant studies. Third, this review did not include gray literature sources such as dissertations, Google Scholar, or preprints, which may have led to publication bias. Fourth, all included studies were from high-income countries, and outcomes may differ in low- and middle-income countries due to contextual and resource variations. Finally, given the rapid pace of technological development in AR and AI, studies published after the search cutoff date were not captured, highlighting the need for future updates.

## Conclusion

At present, the evidence for AR and AI SN assessment and rehabilitation methods remains insufficient to determine their feasibility, effectiveness, ecological validity, and degree of personalization. Future research should focus on developing standardized and scalable frameworks that integrate AR with adaptive AI models to enable personalized and ecologically valid assessment and rehabilitation. Multicenter RCTs are needed to confirm efficacy, optimize hardware configurations, and evaluate long-term functional outcomes.

## Supplemental Material

sj-docx-1-nnr-10.1177_15459683261445440 – Supplemental material for Augmented Reality and Artificial Intelligence for the Assessment and Rehabilitation of Spatial Neglect: A Systematic Review Supplemental material, sj-docx-1-nnr-10.1177_15459683261445440 for Augmented Reality and Artificial Intelligence for the Assessment and Rehabilitation of Spatial Neglect: A Systematic Review by Shaojun Li, Benjamin Chong, Ghazal Mehri-Kakavand, Catherine Shi, Denise Taylor, Allan Fowler, Mark Billinghurst, Monika Harvey and Alan Wang in Neurorehabilitation and Neural Repair

sj-docx-2-nnr-10.1177_15459683261445440 – Supplemental material for Augmented Reality and Artificial Intelligence for the Assessment and Rehabilitation of Spatial Neglect: A Systematic Review Supplemental material, sj-docx-2-nnr-10.1177_15459683261445440 for Augmented Reality and Artificial Intelligence for the Assessment and Rehabilitation of Spatial Neglect: A Systematic Review by Shaojun Li, Benjamin Chong, Ghazal Mehri-Kakavand, Catherine Shi, Denise Taylor, Allan Fowler, Mark Billinghurst, Monika Harvey and Alan Wang in Neurorehabilitation and Neural Repair
