# Supplementary material for: Augmented Reality and Artificial Intelligence for the Assessment and Rehabilitation of Spatial Neglect: A Systematic Review
Source: Neurorehabil Neural Repair. 2026 May 4;40(8):678–92. doi: 10.1177/15459683261445440 (PMC13392187; doi:10.1177/15459683261445440)
Supplement: sj-docx-1-nnr-10.1177_15459683261445440 – Supplemental material for Augmented Reality and Artificial Intelligence for the Assessment and Rehabilitation of Spatial Neglect: A Systematic Review [file sj-docx-1-nnr-10.1177_15459683261445440.docx]

**Supplementary Appendix 1. Database Search Strategy**

| **Database** | **Search String / Keywords Used** | **Search Date** | **Number of Records** |
| --- | --- | --- | --- |
| PudMed | ("unilateral neglect"[Title/Abstract] OR "hemispatial neglect"[Title/Abstract] OR "spatial neglect"[Title/Abstract] OR "hemineglect"[Title/Abstract] OR "hemi-inattention"[Title/Abstract] OR "visuospatial neglect"[Title/Abstract])  AND  ("augmented reality"[Title/Abstract] OR "mixed reality"[Title/Abstract] OR "extended reality"[Title/Abstract] OR AR[Title/Abstract] OR MR[Title/Abstract] OR XR[Title/Abstract] OR "XR system"[Title/Abstract] OR "AR-based"[Title/Abstract] OR "MR-based"[Title/Abstract] OR "XR-based"[Title/Abstract] OR "AR application"[Title/Abstract] OR "XR application"[Title/Abstract] OR "MR application"[Title/Abstract])  AND  (assessment[Title/Abstract] OR diagnosis[Title/Abstract] OR detection[Title/Abstract] OR rehabilitation[Title/Abstract] OR treatment[Title/Abstract] OR therapy[Title/Abstract] OR intervention[Title/Abstract])  AND("Artificial Intelligence"[Title/Abstract] OR "AI"[Title/Abstract] OR "Machine Learning"[Title/Abstract] OR "Deep Learning"[Title/Abstract] OR "Large Language Models"[Title/Abstract] OR "LLM"[Title/Abstract] OR "ChatGPT"[Title/Abstract] OR "Generative AI"[Title/Abstract] OR "Transformer Models"[Title/Abstract])  AND  (assessment[Title/Abstract] OR diagnosis[Title/Abstract] OR detection[Title/Abstract] OR rehabilitation[Title/Abstract] OR treatment[Title/Abstract] OR therapy[Title/Abstract] OR intervention[Title/Abstract]) | 12/8/2025 | 24 |
| Scopus | (TITLE-ABS-KEY("unilateral neglect" OR "hemispatial neglect" OR "spatial neglect" OR "hemineglect" OR "hemi-inattention" OR "visuospatial neglect"))  AND  (TITLE-ABS-KEY("augmented reality" OR "mixed reality" OR "extended reality" OR AR OR MR OR XR OR "XR system*" OR "AR-based" OR "MR-based" OR "XR-based" OR "AR application*" OR "XR application*" OR "MR application*"))  AND  (TITLE-ABS-KEY(assessment OR diagnosis OR detection OR rehabilitation OR treatment OR therapy OR intervention))  AND  (TITLE-ABS-KEY("Artificial Intelligence" OR "AI" OR "Machine Learning" OR "Deep Learning" OR "Large Language Models" OR "LLM" OR "ChatGPT" OR "Generative AI" OR "Transformer Models"))  AND  (TITLE-ABS-KEY(assessment OR diagnosis OR detection OR rehabilitation OR treatment OR therapy OR intervention)) | 12/8/2025 | 47 |
| CINAHL | ("unilateral neglect" OR "hemispatial neglect" OR "spatial neglect" OR "hemineglect" OR "hemi-inattention" OR "visuospatial neglect")  AND  ("augmented reality" OR "AR" OR "mixed reality" OR "MR" OR "extended reality" OR "XR" OR "XR system*" OR "AR-based" OR "XR-based" OR "MR-based" OR "AR application*" OR "XR application*" OR "MR application*")  AND  (assessment OR diagnosis OR detection OR rehabilitation OR treatment OR therapy OR intervention)  AND  ("Artificial Intelligence" OR "AI" OR "Machine Learning" OR "Deep Learning" OR "Large Language Models" OR "LLM" OR "ChatGPT" OR "Generative AI" OR "Transformer Models")  AND  (assessment OR diagnosis OR detection OR rehabilitation OR treatment OR therapy OR intervention) | 12/8/2025 | 19 |
| Web of Science | TS=("unilateral neglect" OR "hemispatial neglect" OR "spatial neglect" OR hemineglect OR "visuospatial neglect")  AND  TS=(gamification OR "serious game*" OR "rehabilitation game*" OR exergame* OR exergaming OR "video game*" OR "game-based therapy")  AND  TS=(assessment OR diagnosis OR detection OR rehabilitation OR treatment OR therapy OR intervention)  AND  TS=("Artificial Intelligence" OR "AI" OR "Machine Learning" OR "Deep Learning" OR "Large Language Models" OR "LLM" OR "ChatGPT" OR "Generative AI" OR "Transformer Models")  AND  TS=(assessment OR diagnosis OR detection OR rehabilitation OR treatment OR therapy OR intervention) | 12/8/2025 | 98 |
| Embase | (unilateral neglect or hemispatial neglect or spatial neglect or hemineglect or hemi-inattention or visuospatial neglect).ti,ab.  AND  (augmented reality or mixed reality or extended reality or XR or AR or MR).ti,ab.  AND  (assessment or diagnosis or detection or rehabilitation or treatment or therapy or intervention).ti,ab.  AND  ("Artificial Intelligence" or "AI" or "Machine Learning" or "Deep Learning" or "Neural Networks" or "Large Language Models" or "LLM" or "ChatGPT" or "Generative AI" or "Transformer Models")  AND  (assessment or diagnosis or detection or rehabilitation or treatment or therapy or intervention)).ti,ab. | 12/8/2025 | 37 |
| IEEE Xplore | ("Document Title":"spatial neglect" OR "Document Title":"hemispatial neglect")  OR  ("Abstract":"spatial neglect" OR "Abstract":"hemispatial neglect"))  AND  (("Document Title":"augmented reality" OR "Document Title":"mixed reality" OR "Document Title":"extended reality")  OR  ("Abstract":"augmented reality" OR "Abstract":"mixed reality" OR "Abstract":"extended reality"))  AND  ("Artificial Intelligence" OR "AI" OR "Machine Learning" OR "Deep Learning" OR "Large Language Models" OR "LLM" OR "ChatGPT" OR "Generative AI" OR "Transformer Models")  AND  (assessment OR diagnosis OR detection OR rehabilitation OR treatment OR therapy OR intervention) | 12/8/2025 | 17 |
| ACM Digital Library | ("Augmented Reality"[MeSH] OR "Augmented Reality" OR "AR" OR "Mixed Reality" OR "Extended Reality" OR "XR" OR "Holographic Display" OR "MR") AND ("Spatial Neglect"[MeSH] OR "Spatial Neglect" OR "Hemineglect" OR "Unilateral Neglect" OR "Visuospatial Neglect" OR "Unilateral Spatial Neglect" OR "Hemispatial Neglect" OR "Left-Sided Neglect" OR "Right-Sided Neglect") AND ("Rehabilitation"[MeSH] OR "Rehabilitation" OR "Cognitive Training" OR "Motor Training" OR "Neurorehabilitation" OR "Telerehabilitation" OR "Virtual Rehabilitation" OR "Occupational Therapy" OR "Physical Therapy") AND  ("Artificial Intelligence" OR "AI" OR "Machine Learning" OR "Deep Learning" OR "Large Language Models" OR "LLM" OR "ChatGPT" OR "Generative AI" OR "Transformer Models")  AND  (assessment OR diagnosis OR detection OR rehabilitation OR treatment OR therapy OR intervention) | 12/8/2025 | 26 |
| Total Paper |  |  | 268 |
| Total After remove duplicates |  |  | 172 |
| Total after Title & Abstract Screening |  |  | 32 |
| Total after full paper screening |  |  | 15 |
