# Supplementary material for: Augmented Reality and Artificial Intelligence for the Assessment and Rehabilitation of Spatial Neglect: A Systematic Review
Source: Neurorehabil Neural Repair. 2026 May 4;40(8):678–92. doi: 10.1177/15459683261445440 (PMC13392187; doi:10.1177/15459683261445440)
Supplement: sj-docx-2-nnr-10.1177_15459683261445440 – Supplemental material for Augmented Reality and Artificial Intelligence for the Assessment and Rehabilitation of Spatial Neglect: A Systematic Review [file sj-docx-2-nnr-10.1177_15459683261445440.docx]

Supplementary Appendix 2.a.Characteristics of the included rehabilitation and assessment studies.

| **Study** | **Study Design** | **Sample Size** | **Age (Mean ± SD or Range)** | **Sex (M/F)** | **Stroke Stage & Time & Etiology** | **Lesion Location** |
| --- | --- | --- | --- | --- | --- | --- |
| Mak et al., 2022, United States | Pilot Study | 5 with stroke and SN and 5 with stroke without SN (WSN) | SN (mean+-SD  61.4+-18.6)  WSN: (mean+-SD  54.4+-23.1) | SN:2M,3F  WSN:4M,1F | SN:151 +-307 (4 early Subacute, 1 chronic)  WSN:1238+- 1130 (1 early Subacute, 4 chronic)  NR | SN:4 right,1 left  WSN:2 right, 3 left |
| Takazawa et al., 2022, Japan | Feasibility single-case study | N=1(left USN) | 52 | M | late subacute; 124 days post-onset  Hemorrhage | patient had left USN |
| Kocanaogullari et al., United States | Feasibility study | 5 SN and 5 WSN | SN:(mean+-SD  67.4+-11.5)  WSN:(mean+-SD  53.4+-19.8) | SN:2M,3F  WSN:3M,2F | SN: 208 +-288 (2 acute, 2 chronic,1 nr)  WSN: 1239+- 1010(1 early Subacute, 4 chronic)  NR | SN: Right=4, Left=1; WSN: Right=2, Left=3 |
| Stammler et al., 2023 Germany | Feasibility study | 10 SN and 10 healthy older adults | 61.3 ± 15.2 years (patients)  66 ± 11.9 years (healthy) | SN:8 M,2F  healthy:NR | Mean 138.4 ± 192.1 days  7 early subacute,  1  late subacute,  2 chronic  ischaemic n=5,haemorrhagic n=5 | Right hemisphere 10 |
| Stammler et al., 2023 Germany | RCT | N=20 SN(10 Negami, 10 standard) | Negami 61.3 ± 15.2; Standard 60.7 ± 12.45 | Negami 8M, 2F; Standard 6M,4F | negami:138.4 ± (192.1)  standard: 84 ± 33.86  Negami 5 infarcts/5 hemorrhages; Standard 4 infarcts/6 hemorrhages | Right hemisphere 20 |
| Bakker et al., 2020, Netherlands | Design-based research (DBR) study | 7 SN and 8 OTs and 7 other HCPs | NR | NR | NR | NR |
| Stammler et al., 2024 Germany | Cross-sectional diagnostic accuracy study | 20 SN and 20 Healthy controls | Patients 59.6 ± 14.6; Controls 60.1 ± 13.4 | Patients 15M, 5F; Controls 10M, 10F | patients 145.7 ± 185.5 days post-stroke  8 infarcts / 12 hemorrhages | Right hemisphere 20 |
| Liang et al. (2010), United Kingdom | Observational case-control study | Training: 33 SN+ 110 stroke controls; Testing: 19 SN+ 27 stroke controls | > 60 year old | NR | NR | NR |
| De Boi et al. (2024), Belgium | Cross-sectional diagnostic accuracy study | HC15, WSN 13, SN 10 | HC 55.4 ± 24.4; WSN 63.2 ± 18.2; SN 67.0 ± 19.3 | HC 10M,5F; WSN 7M,6F; SN 4M,6F | NR  Ischemic,  hemorrhagic | Right hemisphere |
| Donisi et al. (2021), Italy | Cross-sectional diagnostic accuracy study | SN 11, WSN 24 | 59.89 ± 14.51 (overall) | 20M,15F | NR,  Haemorrhagic 19 Ischaemic 16 | Right 19 / Left 16; all USN on right |
| Kocanaogullari et al. (2020), United States | Cross-sectional diagnostic accuracy study | SN=5, WSN=6 | NR | SN 3M,2F,  WSN 3M,3F | SN: 64.6 ± 10.4:  1 acute, 2 early subacute, 1 late subacute  WSN: 68.2 ± 7.4:  1 acute, 5 early  NR | WSN:Right=1,Left=4,Bilateral=1; SN: Right=4,Left=1 |
| Rosenzopf et al. (2023), Germany | Cross-sectional diagnostic accuracy study | SN 12, WSN 8 | SN 71.0 ± 8.3; WSN 56.9 ± 15.9 | SN 8M,4F; WSN 7M,1F; | Acute: tested 6.4 ± 4.5 days post-stroke 19; Chronic: 32 months 1;  Ischaemic 20 | Right hemisphere |
| Belger et al. (2023), Germany | Cross-sectional diagnostic accuracy study | SN 20, WSN 19, HC 20; | SN 60.00 ± 6.87; WSN 56.82 ± 10.03; HC 59.05 ± 9.52 | SN: 15M, 5F; WSN: 10M,9F; HC: 13M,7F | Chronic; SN 37.80 ± 32.99, WSN 50.00 ± 49.80  NR | Right hemisphere |
| Franceschiello et al., 2022, Switzerland and France | Cross-sectional diagnostic accuracy study | SN 15; HC 9 | SN: ~58 (range 45–69); HC: age-matched | SN: 9M, 6F | Chronic; 306.5 +-201.6  Ischemic 11,  Hemorrhagic 3,  Mixed 1; | Right hemisphere |
| Kim et al., 2021, Republic of Korea | Cross-sectional diagnostic accuracy study | SN 19, WSN 22, HC 22; | SN: 54.32±7.40; WSN: 49.23±9.99; HC:  45.41±17.82 | SN 14F,5M; WSN 17F, 5M; HC 10M,12F; | ≥3 months post-stroke; months since stroke: SN 11.79±12.37, WSN 10.57±10.96  NR | Right hemisphere |

Supplementary Appendix 2.b. Representativeness, Diagnostic Methods, and Group Comparability of Included Studies

| Study | Representativeness | SN Diagnosis Method(s) | Control group stroke severity comparability |
| --- | --- | --- | --- |
| Mak et al., 2022, United States | Convenience, community + inpatient rehab; screened 226, enrolled 10; not consecutive | Participants completed the BIT-C. If any BIT-C subtests scores were below each subtest’s cutoff or the total score was below 129, the participant was categorized as SN. | Same sources (community + inpatient rehab); age/sex match NR; stroke severity (NIHSS/mRS) NR; BIT-C used to define groups (thus not comparable on neglect severity by design). |
| Takazawa et al., 2022, Japan | Convenience (single-patient validity test at Sonodakai Rehabilitation Hospital) | CBS used for outcome (diagnostic route NR) | NA (no control group) |
| Kocanaogullari et al., United States | Convenience; two research datasets (AREEN & CBBCI), IRB-approved | Participants completed the BIT-C. If any BIT-C subtests scores were below each subtest’s cutoff or the total score was below 129, the participant was categorized as SN. | Same sources (community + inpatient rehab); age/sex match NR; stroke severity (NIHSS/mRS) NR; BIT-C used to define groups (thus not comparable on neglect severity by design). |
| Stammler et al., 2023 Germany | Convenience, single-site inpatient rehab; non-consecutive | Inclusion required neglect on ≥2 of 4 tablet-based tests: Letter Cancellation, Bells, Copying, Line Bisection; cutoffs: CoC ≥ 0.08, Copying score > 1, EWB ≥ 0.07 | NR |
| Stammler et al., 2023 Germany | Consecutive inclusion of all eligible patients at 3 sites; randomized by admission order (time criterion) | Must meet neglect on ≥2 of 4 tests (Letter Cancellation, Bells, Copying, Line Bisection) with cutoffs: CoC ≥ 0.08, Copying > 1, EWB ≥ 0.07 | NIHSS/mRS: NR; groups comparable at baseline on age, sex, and post-stroke interval |
| Bakker et al., 2020, Netherlands | Convenience, 3 skilled-nursing geriatric rehab facilities; co-creation sample | CBS or prior hospital neuropsychological tests for VSN (per inclusion) | NR |
| Stammler et al., 2024 Germany | Convenience, 3 rehab facilities | Neglect required ≥2/4 tests positive with cutoffs: Letter Cancellation & Bells CoC ≥ 0.08; Copying > 1; Line Bisection EWB ≥ 0.07. | NR (healthy controls only); NIHSS/mRS NR |
| Liang et al. (2010), United Kingdom | Convenience sample of post-stroke patients; not stated as consecutive | BIT (Conventional subset); total <130 = neglect; also mapped to a four-point scale for severity | NIHSS/mRS: NR; stroke controls defined via BIT (severity matching NR) |
| De Boi et al. (2024), Belgium | Convenience; single rehab site; recruitment Oct 2020–Sep 2021 | BHT, LBT, VSTT; neglect if ≥ 2/3 positive | NIHSS/mRS: NR; baseline severity comparability NR |
| Donisi et al. (2021), Italy | Convenience, single inpatient neurorehb unit; | NR（labels = clinical USN vs non-USN; specific test not stated | NIHSS/mRS: NR; MoCA/FIM/BI showed no baseline differences between groups (p>0.05) |
| Kocanaogullari et al. (2020), United States | Convenience (single-center research sample) | BIT (Conventional); SN if BIT total<129 or >1 subtest below cutoff | NIHSS/mRS: NR |
| Rosenzopf et al. (2023), Germany | Consecutive/continuous admissions, single center | Letters & Bells cancellation (CoC, cutoffs ±0.083/±0.081) + Copying; SN if ≥2/3 pathological | NIHSS/mRS NR; groups not severity-matched (age differs) |
| Belger et al. (2023), Germany | Convenience; out-patient rehab clinic + database; not consecutive | Clinical letters + neuropsych battery (CBS; NET line crossing/copying/line bisection/picture scanning; SNT CoC with cutoffs 0.081 single / 0.118 dual); USN− required no history of neglect and normal battery | NIHSS/mRS NR; age not significantly different; other baseline tests differ in places |
| Franceschiello et al., 2022, Switzerland and France | Convenience (recruited from prior study/database) | Paper-and-pencil battery with explicit cutoffs: Bells (left–right omission difference > 2); Letter cancellation (≥1 target undetected in each hemifield); Line bisection (deviation beyond −7.3 mm leftward or +6.5 mm rightward on a 200-mm line); Landscape drawing (any error > 0); Reading (left–right omission difference > 0). Patients diagnosed as neglect if pathological per these rules. | NR |
| Kim et al., 2021, Republic of Korea | Consecutive stroke recruitment (single-center rehabilitation hospital); healthy controls recruited via convenience sampling. | HSN was diagnosed if any one of the following criteria was met: LBT < 7, SCT < 51, or CBS > 1 (composite reference). | NIHSS/mRS not reported; no significant between-group differences in age, sex, or time from stroke onset to assessment (statistical comparison provided); severity matching details not reported. |

Supplementary Appendix 2.c. Characteristics and key findings of the included AI assessment studies

| Study | Technology & model (platform + algorithm) | Task / Label | Input & Features | Validation | Hardware & setup | Metrics reported (test/CV) | Key result(s) |
| --- | --- | --- | --- | --- | --- | --- | --- |
| Mak et al., 2022 | AR–EEG; 1.SN detection:bandpower ratio → logistic regression; 2.Response prediction::CSP → RDA + KDE  baselines: LDA/QDA, Naive Bayes, MLP, RF, AdaBoost | SN detection (SN vs WSN via BIT-C); response prediction (fast vs slow RT as proxy of attended/neglected) | Bandpower ratios (ipsi/contra) δ–γ for SN detection; CSP features (~16-dim) for response prediction | Subject-wise 10-fold CV; training-only fitting for all preprocessing/feature selection; 10 pts; 2 sessions/p; 72 median-labeled targets/session; fast/slow stratified in training. | AR: Microsoft HoloLens (1st-gen) + Bluetooth clicker; PC as control.  EEG: g.USBamp; 16 electrodes (Fp1, Fp2, F3, F4, Fz, FC1, FC2, Cz, P1, P2, C1, C2, CP3, CP4, O1, O2); Fpz ground, left mastoid reference; 256 Hz sampling.  Sync/latency: ArduinoBLE bridge (PC→HoloLens via BLE, PC→amp via cable); measured two-way BLE delay ≈ 75 ms, alignment uses Dm = 37.5 ms (half RTT) for trigger correction. | SN detection: test AUC = 0.832; response prediction: RDA+KDE best vs baselines (numeric NR) | Predictive EEG loci: frontal-central δ/α, frontal-parietal θ, Fp1 β, left-frontal γ; fast-response ratio correlated with BIT-C (R≈0.81) |
| Kocanaogullari et al., 2021 | EEGNet (modified): conv→depthwise→separable conv, ELU, avg-pool, dropout; trained on other subjects then fine-tuned (100 epochs, Adam 1e-4) on the target subject | Classify slow vs fast response targets (proxy for neglected vs attended); labels via Otsu on the median of 3 RTs per grid cell with within-cell majority vote | 700 ms post-target epoch @ 256 Hz; 2–60 Hz band-pass + 58–62 Hz notch; first 200 ms baseline correction; 14 channels (common subset) | Subject-wise transfer: evaluate on held-out subject (before fine-tuning), then fine-tune on that subject and re-evaluate | 1AREEN: AR headset task; CBBCI: screen-based version of the same paradigm; EEG 14-channel montage @256 Hz | Per-subject accuracy (Table III): before 6.5–64.3% → after 65.4–98.6%; fine-tuning time ≈ 60–129 s (per subject) | Fine-tuning markedly boosts per-subject accuracy within ~1–2 min, supporting rapid personalization for clinical use |
| Liang et al. (2010) | Computer-based pen-tablet assessment; per-task BLR models with rule-based feature selection; unweighted/weighted voting; weighted linear model combining cancellation (weight 130) + drawing (regression) to approximate BIT; baseline linear regression with automated selection | Automated scoring of computer-based drawing & cancellation tasks to (i) classify neglect vs stroke control (binary), and (ii) approximate BIT on four-point and continuous scales | 14 tasks + 3 derived; sampling 100 Hz; 57 cancellation-task features & 35 drawing-task features (e.g., ON-OFF-R, PEN-DIS, X-CENTRE, TIME-PER-CAN, TOTAL-LR/MOVE-LR); features normalized and combined per task | Yes — subject-independent split: train on Dataset-1 (33+110), test on unseen Dataset-2 (19+27); prior LOOCV discussed but final evaluation uses disjoint sets | WACOM Intuos2 graphics tablet + cordless inking pen; paper overlays (single figure per sheet); PC logging at 100 Hz | Weighted linear model: AER = 7.5% (FAR 3.8%, FRR 11%); agreement with BIT ≈ 92.5% (binary). Kappa (four-point) and Spearman (continuous) significant (values NR). Optimal voting (7 tasks) AER ≈ 14.5% | Best performer = weighted linear model; achieves significant agreement with BIT across binary/four-point/continuous formats; fewer overlays (≈6) and shorter admin time (avg < 3 min; max 10 min) than full BIT; captures dynamic drawing features objectively |
| De Boi et al. (2024) | Unity 2020.2.5f1 VR app on Pico Neo 2 Eye HMD (built-in Tobii eye-tracking); Gaussian Process Regression maps 2D field-of-view → search time; active learning (uncertainty sampling) to place next stimulus | VSN detection via VR-derived indices (SAM of gaze/head/eye: GR/HR/ER); labels from clinical gold standard (BHT/LBT/VSTT, neglect = ≥ 2/3 positive) | Head & eye angles at ~10 Hz; compute max left/right angles; SAM = (right − left) on levels T2/T3 (near/table) & P2/P3 (far/playground) | Case–control ROC on independent groups (no CV); test–retest (≈ 1 week) for intra-rater reliability | Pico Neo 2 Eye (6-DoF, ~101° FOV, 4K); seated/wheelchair, trunk restrained; calibration; gaze-hold until on-screen circle completes | ROC: multiple parameters AUC > 0.80; example cut-offs—SAM GR P2 ≥ 0.00585 (sens ≈ 0.80), SAM GR P3 ≥ 0.0218 (sens ≈ 0.70), SAM HR P3 ≥ 7.4°; Intra-rater: 10/12 measures NS, 2 small learning effects; SSQ: no moderate–high cybersickness | VR test more sensitive than paper-and-pencil; good intra-rater reliability; GP heatmaps delineate neglect boundary → basis for border-cueing therapy logic (treatment not trialed here) |
| Donisi et al. (2021) | Tree-based ML: Decision Tree (Gini, no pruning), Random Forest (Info Gain Ratio), Rotation Forest (J48 base), AdaBoost (decision stumps), Gradient Boosting (max depth=4, LR=0.1); wrapper backward feature elimination; feature importance by Information Gain; SMOTE for class balance | Binary classification: USN vs non-USN in stroke | Six clinical features: MoCA, FIM, Barthel Index (BI), aetiology, site of brain lesion, lower-limb hemiparesis (Y/N) | Yes — 10-fold CV on N=35; SMOTE used（application within folds not specified）; no external test set | Clinical record–based; implementation in KNIME 4.1.3(stats in GraphPad Prism 9) | Best (Random Forest): Acc 0.92, Sens 0.83, Spec 1.00, Prec 1.00, AUC 0.95; others: DT AUC 0.87, RotF 0.91, AdaBoost 0.92, GB 0.92 | Site of lesion = most informative feature; MoCA/FIM/BI carried little/no information; only lesion-side differed at baseline; small N and use of SMOTE noted as limitations |
| Kocanaogullari et al. (2020) | Screen-based Starry Night paradigm + EEGNet (CNN); temporal conv → depthwise & separable conv → avg-pool → dropout → softmax; ~1.4k params; Adam (1e−3), 100 epochs, batch 16 | SN vs WSN classification using EEG segments linked to slow-response (neglected) targets (ground truth from calibration via Otsu threshold on RT) | 16-ch EEG @256 Hz; 700 ms target-locked epochs; 2–62 Hz Butterworth + 58–62 Hz notch; last 200 ms spectral baseline correction; per-channel min–max normalization | 10-fold CV; subject-wise independence not specified (segment-level folds) | Seated 114 cm from screen (FOV ≈ 17.23°×9.74°); 8×8 grid; 192 targets (66 ms each), inter-target 700–2200 ms; distractors 50–250 ms; 16 electrodes (Fp1, Fp2, F3, F4, Fz, FC1, FC2, Cz, P1, P2, C1, C2, CP3, CP4, P5, P6) | 10-fold CV (test): Acc 89.73%, Spec 89.34%, Sens 86.97%, F1 0.8934; AUC: NR | Most informative electrodes included Cz, P1, F3; paradigm designed to enable future FOV severity estimation |
| Rosenzopf et al. (2023) | Computerized cancellation (MATLAB 2016b + Psychtoolbox) on 27″ capacitive touchscreen; SVM (linear/RBF), nested LOSO grid-search; MICE for missing data | Binary Neglect vs No-neglect (labels from clinical screening: pathologic on ≥2/3 [Letters, Bells, Copying]) | Process features from digital tests: search speed (#targets/sec), search distance (mean Euclidean step), search strategy (row/column-wise pattern via mean x/y step) | Leave-One-Subject-Out CV; hyper-parameters tuned via nested LOSO | 3 sizes on same 27″ display: TS-small 260.28×173.52 mm, TS-medium 297×210 mm (A4), TS-large 597.6×336.2 mm; flat on table; stylus (Adonit Dash 2) | Balanced accuracy (test): Bells 97.92%, Letters 88.19%; accuracy independent of screen size; AUC NR | CoC robust to format/size (paper vs digital; small/medium/large). Process features differ by group (neglect: ↓speed, ↑distance, more “strategic”). SVM reliably classifies neglect across sizes; potential early-stop diagnosis when CoC unavailable. |
| Belger et al. (2023) | Immersive virtual road-crossing (iVRoad) + Random Forest and CART; feature filter via overfitting CART → sequential backward search with RF (mlr3/ranger) | 3-class classification: USN+ vs USN− vs Healthy; labels from clinical diagnosis (medical letters) and battery (CBS/NET/SNT with CoC cutoffs) | 113 VR features across: temporal（reaction time, decision time, letter-insertion time, total experimental time）, performance（errors）, head movement（yaw, roll, head-switch count）. Top-6：SD(reaction-time crossing); SD(reaction-time crossing when cars from left); Mean(head-switches when cars from right); Min(letter-insertion time for left box); Total experimental time; Mean(left-yaw when cars from left) | Repeated 10×10-fold CV, stratified by group, participant-level vectors; no external test set | HTC Vive Pro Eye (≈110° FOV, 1440×1600/eye); 24 trials/subject; factors：mailbox side L/R, traffic 30/50 km·h⁻¹, gaps 6.5/7.5 s; seated, trunk free | Estimated test accuracy 76.8%（repeated CV）; training acc 96.6%; AUC NR | Side-specific temporal + head-movement features are most predictive; VR-ML > single conventional tests（sens 0–35%; aggregated rule ≈ 65%）; no external validation |
| Franceschiello et al., 2022 | Traditional ML (SVM, Random Forest, AdaBoost) and CNNs (1D & 2D) on eye-movement trajectories; standardized preprocessing pipeline | Binary classification: Neglect vs Healthy (labels from clinical diagnosis & battery) | Left-hemifield trials only; per trial 300 Hz gaze trajectories; vectors standardized（first 3 s of search after 3 s cue）; x and y projections (length≈1001) for 1D models; 2D image / 2D tensor for CNN variants | 5-fold CV at participant level, repeated 10 runs with new splits each run; majority vote to assign subject label | Tobii TX300 eye-tracker (300 Hz); visual-search task with central cue 3000 ms then target until response or 6000 ms; 176 trials/session; joystick up/down responses | Across runs (x-projection): AUC 0.83–0.86; typical values——1D-CNN AUC≈0.85, Acc≈86%；SVM AUC≈0.84, Acc≈88%；RF AUC≈0.85, Acc≈88%；AdaBoost AUC≈0.86, Acc≈89%（2D-image CNN significantly worse; 2D-vector ≈ 1D-CNN） | The x-coordinate was more informative than the y-coordinate; the 2D-image CNN performed worse than the 1D-CNN (Wilcoxon p ≈ 0.01). The 1D-CNN confidence score correlated with SLF3 fractional anisotropy (ρ ≈ −0.77, p = 0.003, Bonferroni-corrected) and with the number of omissions on the left side in the Bells test (ρ ≈ 0.55, p = 0.033). A reusable preprocessing codebase and an open dataset were provided. |
| Kim et al., 2021 | VR FOPR test on HMD; SVM with RBF kernel; hyper-parameter grid search (C, gamma); feature normalization | Binary classification: hemispatial neglect (HSN) vs non-HSN stroke (healthy controls used for group comparisons, not the classifier) | FOP (Field of Perception): success rate and reaction time with head fixed, 30 trials; FOR (Field of Regard): success rate and reaction time with free head rotation, 90 trials. Features computed for Left and Right, and for Near and Far space; also combined “both-spaces” indices | 5-fold cross-validation at the participant level (subject-wise splits); no external test set | Oculus Rift DK1 HMD (1280×800, 3-DoF head sensor); Vizard 4.0 on Windows; mouse responses. Total assessment ~10–20 min | Cross-validated accuracy, sensitivity, specificity, PPV, NPV | Left-space features performed best (e.g., FOP-Left-Near reached 100% accuracy; most Left conditions ≥83.3%). Right-space performance was lower (≈50–66.7%). FOP/FOR patterns (lower success rate, longer reaction time) aligned with clinical neglect severity; Bland–Altman analyses showed good agreement with LBT, SCT, and CBS measures. |

Supplementary Appendix 2.d. Characteristics and key findings of the included AR rehabilitation and assessment studies.

| Study | **Device / Platform** | **Task paradigm & setting** | **Guidance modality** | **Dosage (duration × freq × weeks)** | **Outcome measures** | **Pre to Post** | **Adherence / AEs** | **Based on traditional method** |
| --- | --- | --- | --- | --- | --- | --- | --- | --- |
| Takazawa et al., 2022 | HoloLens 2 + PC; Unity 2019.4.23 | Daily-life object search with ambulation; 16 holograms per lap (8 upper, 8 lower), randomized; gaze logging | Second-lap cue for missed left targets (chime + red ball); on-device counts/time feedback | 20 min/session × daily × 2 weeks | CBS; gaze-left ratio (GR%) | CBS 12 → 6; GR% 40.1 → 74.9 | Completed; no adverse events | Visual scanning training / cueing-based visual exploration in ADL-like environments |
| Stammler et al., 2023 | iPad Pro 12.9″ (3rd gen); Xamarin (C#) + ARKit; cloud sync via SQLite → SQL Server + Azure Blob | Two tasks in ward room, seated: Task A “follow the bird” (keep bird in on-screen circle by turning gaze/head/trunk); Task B “find the bird” (therapist hides bird within angle range; patient searches) | Visual crosshair; optional blue compass cue; auditory tones for success/holding | 20–25 min/session × 5×/week × 2 weeks; start easy, advance after 3 successes; downgrade after 2 failures | SUS, SSQ, PGTQ; patient ratings (satisfaction, motivation, fun) | Neglect severity: NA (not reported).  Usability/experience (post-only): Healthy n=10 — SUS mean 4.3 ± 0.6/5, SSQ mean 0.8 ± 1.32; PGTQ: motivation 6.1 ± 0.7, entertainment 6.5 ± 0.9, frustration 1.5 ± 0.7, challenge 4.1 ± 1.7. Patients n=10 — all recommended the app; ratings for satisfaction/motivation/fun were within the top two categories. | Low cybersickness; 3/10 (30%) reported tablet weight discomfort; completion NR | Visual exploration / scanning training with contralesional rotation of eyes, head, and trunk |
| Stammler et al., 2023 | iPad Pro 12.9″ (3rd gen); Negami app (AR) | Ward-based training while seated/standing: Task A “Follow the bird” (keep virtual bird in target circle by turning eyes/head/trunk) → Task B “Find the bird” (therapist hides bird; active search)  trandinal：Standard visual exploration/scanning therapy: 5 sessions/week; smooth pursuit eye movement training (dot clouds moving 5–10°/s from ipsilesional to contralesional) plus reading/copying, picture description, and search tasks to promote active contralesional orienting. | On-screen target circle; auditory cues; level increases after 3 successes, decreases after 2 failures | ≈25 min/session × 5 sessions/week × 2 weeks (10 sessions total) | Letter Cancellation, Bells, Copying, Line Bisection, “Exploration Test” (weekly E1–E5; Negami group additional follow-up E6 at 1–2 mo) | Both groups improved, but Negami > Standard: at E4/E5, Negami showed significantly greater gains in Bells, Copying, Exploration (and at E5 also Letter Cancellation); effects present after week-1 and maintained at 1–2 mo in Negami group | Training completed per protocol; difficulty advanced for most patients; adverse events NR | Visual exploration / scanning training with coordinated eye–head–trunk orientation to contralesional space |
| Bakker et al., 2020 | Microsoft HoloLens (1st-gen); Unity 5.6.2, C#, Windows 10 Mobile; local dedicated router; head-movement control; voice prompt (“start”) | Virtual museum: search for virtual “paintings” projected on walls; encourages scanning while moving (walking/wheelchair); object contrast enhanced (luminous gold frame) | Direct in-game audio feedback when a painting is overlooked (prompt originates from side of missed target); video intro to task | Single session | Qualitative observations (fly-on-the-wall), think-aloud sessions, semi-structured interviews; questionnaire of OTs/HCPs; peer examination | Neglect severity: NA (no quantitative pre–post outcomes).  Usability/qualitative (post-only): Observational fly-on-the-wall and think-aloud plus therapist interviews/questionnaires informed design choices: promote independent mobility with systematic visual scanning; use same-side audio cues for missed targets with graded cueing; increase target contrast/visibility; simplify interactions; fade cues over time to foster internalized strategies. No standardized satisfaction scores reported. | AEs NR; noted usability adjustments (fit of HoloLens; contrast tuning) | Visual Scanning Training (VST) principles: systematic visual exploration, eye/head rotation to neglected side, repetition, direct feedback, cue reduction |
| Stammler et al., 2024 | iPad Pro 12.9″ (3rd gen); | Seated/wheelchair; familiarization with target at +10° then −10°; test phase: no target hidden, record 30 s free tablet movements (arm + trunk rotation) to capture exploration bias | No cues in test phase (familiarization only shows bird); app outputs dwell-time (2° bins) & mean horizontal exploration angle | Single assessment, ~3 min execution time | Diagnostic accuracy: AUC = 0.89; optimal cut-off = 9.0° right; sensitivity = 0.85, specificity ≈ 1.00; correlations with legacy tests: Letter Cancellation r = 0.56, Bells r = 0.49, Copying r = 0.41, Line Bisection r = 0.22 | NA (assessment-only; no pre–post therapy change) | Completed per protocol; AEs NR; tablet-based AR cited as time-efficient and patient-friendly | Free exploration test |

Supplementary Appendix 2.e. Ecological Validity evaluations.

| Study | Environment | Stimulus | Response | Body | Mind | Total |
| --- | --- | --- | --- | --- | --- | --- |
| Mak et al., 2022 | 0 | 1 | 0 | 1 | 0 | 2 |
| Takazawa et al., 2022 | 1 | 1 | 2 | 2 | 2 | 8 |
| Kocanaogullari et al., 2021 | 0 | 1 | 0 | 1 | 0 | 2 |
| Stammler et al., 2023 | 2 | 1 | 1 | 2 | 1 | 7 |
| Stammler et al., 2023 | 2 | 1 | 1 | 2 | 1 | 7 |
| Bakker et al., 2020 | 2 | 1 | 2 | 2 | 2 | 8 |
| Stammler et al., 2024 | 2 | 1 | 1 | 2 | 2 | 8 |
| Liang et al. (2010) | 0 | 1 | 0 | 1 | 0 | 2 |
| De Boi et al. (2024) | 1 | 1 | 1 | 1 | 1 | 5 |
| Donisi et al. (2021) | 0 | 1 | 0 | 1 | 0 | 2 |
| Kocanaogullari et al. (2020) | 0 | 1 | 0 | 1 | 0 | 2 |
| Kim et al., 2021 | 1 | 1 | 1 | 2 | 1 | 6 |
| Franceschiello et al., 2022 | 1 | 1 | 1 | 1 | 1 | 5 |
| Rosenzopf et al. (2023) | 0 | 1 | 0 | 1 | 0 | 2 |
| Belger et al. (2023) | 1 | 1 | 1 | 1 | 2 | 6 |
